# Comparison of the diagnostic performance of 64-slice computed tomography coronary angiography in diabetic and non-diabetic patients with suspected coronary artery disease

**DOI:** 10.1186/1475-2840-9-80

**Published:** 2010-11-29

**Authors:** Daniele Andreini, Gianluca Pontone, Antonio L Bartorelli, Piergiuseppe Agostoni, Saima Mushtaq, Laura Antonioli, Sarah Cortinovis, Mauro Canestrari, Andrea Annoni, Giovanni Ballerini, Cesare Fiorentini, Mauro Pepi

**Affiliations:** 1 Centro Cardiologico Monzino, IRCCS, Department of Cardiovascular Sciences, University of Milan, Milan, Italy; 2 ASUR Marche, Ospedale Santa Croce, Fano, Italy

## Abstract

**Background:**

Diabetics have high prevalence of subclinical coronary artery disease (CAD) with typical characteristics (diffuse disease, large calcifications). Although 64-slice multidetector computed tomography (MDCT) coronary angiography has high diagnostic accuracy to detect CAD, its diagnostic performance in diabetics with suspected CAD is unknown. To compare the diagnostic performance of 64-slice MDCT between diabetics and non-diabetics with suspected CAD scheduled for invasive coronary angiography (ICA).

**Methods:**

We enrolled one hundred and five diabetic patients (92 men, age 65 +/- 9 years, Group 1) and 105 non-diabetic patients (63 men, age 63+/-5 years, Group 2) with indication to ICA for suspected CAD undergoing coronary 64-slice MDCT before ICA.

**Results:**

In Group 1, the overall feasibility of coronary artery visualization was 93.8%. The most frequent artifact was blooming due to large coronary calcifications (54 artifacts, 67%). In Group 2, the overall feasibility was significantly higher vs. Group 1 (97%, p < 0.0001). In Group 1, the segment-based analysis showed a MDCT sensibility, specificity, positive predictive value, negative predictive value and accuracy for the detection of ≥50% luminal narrowing of 77%, 90%, 70%, 93% and 87%, respectively. In Group 2, all these parameters were significantly higher vs. Group 1. In the patient-based analysis, specificity, negative predictive value and accuracy were significantly lower in Group 1 vs. Group 2.

**Conclusions:**

Although MDCT has high sensitivity for early identification of significant CAD in diabetics, its diagnostic performance is significantly reduced in these patients as compared to non-diabetics with similar clinical characteristics.

## Background

Glucose intolerance and diabetes mellitus (DM) are associated with premature atherosclerosis [1] and increased risk for coronary artery disease (CAD). Moreover, traditional cardiovascular risk factors such as hypertension, dyslipidemia and obesity cluster in patients with DM [2]. All these factors may explain why diabetics have accelerated progression and, often, more aggressive and diffuse development of coronary atherosclerosis in frequently smaller native vessels [3] and experience higher morbidity and mortality. Moreover, CAD diagnosis may be missed or delayed in these patients since the typical symptoms are often masked despite diffuse multi-vessel coronary atherosclerosis is frequently present. Therefore, there is a clear clinical need to detect CAD at an early stage in DM patients who are at risk of both fatal and non fatal cardiac events before the onset of symptoms.

Sixty-four-slice multidetector computed tomography (MDCT) coronary angiography is currently considered a reliable diagnostic method for the evaluation of patients with known or suspected CAD with high diagnostic performance for the detection of significant coronary stenosis [4]. Moreover, several studies demonstrated its ability to detect and differentiate calcified and noncalcified coronary atherosclerotic plaques noninvasively [5]. Iwasaki et al. demonstrated that the prevalence of subclinical atherosclerosis in asymptomatic DM patients assessed with 64-slice MDCT is higher than that observed in asymptomatic non-diabetic patients. Indeed, coronary plaques and significant coronary stenoses were found in 68% and 16% of non-diabetics as compared to 91% and 33% of diabetics [6]. However, only one study demonstrated that MDCT may have a prognostic role in DM patients, showing a significantly lower event-free survival in them as compared to a control population and a close correlation between the presence of significant CAD at MDCT and CAD-related events [7]. Disappointingly, the diagnostic accuracy of MDCT in diabetics can be influenced by two main problems: the increased pre-test likelihood of CAD that affects lower diagnostic performance of MDCT [8,9], and the coronary artery lesions characteristically located in smaller vessels [10] and more frequently presenting extensive calcifications [3,6,11]. These are the main causes of unevaluability of coronary arteries at MDCT [12]. As a result, the clinical role of MDCT coronary angiography as a non-invasive diagnostic tool in diabetics for detecting hemodynamically relevant stenoses before they become clinically evident remains to be determined. Therefore, the aim of this study was to compare the diagnostic performance of 64-slice MDCT between DM patients and a control population with suspected but unknown CAD scheduled for invasive coronary angiography (ICA).

## Methods

### Study population

One hundred and sixteen consecutive diabetic patients (Group 1) and 115 consecutive non-diabetic patients, who were referred for ICA for suspected CAD because of chest pain and/or inconclusive stress test, were enrolled from January 2007 to December 2008 (Table 1). Patients were classified as affected by DM if they carried an established diagnosis of this ailment made by a physician and/or were receiving treatment with insulin or oral hypoglycemic agents. Exclusion criteria were previous ICA, contraindication to the administration of iodine-based contrast agents, pregnancy, history of known CAD, impaired renal function (creatinine clearance <60 ml/min), inability to sustain a 15-second breath hold and cardiac arrhythmias. Based on these exclusion criteria, 21 patients were not enrolled in the study because of inability to sustain the breath hold (3 in Group 1, 2 in Group 2), cardiac arrhythmias (6 in Group 1, 5 in Group 2) and impaired renal function (2 in Group 1, 3 in Group 2). Thus, the analytic study population consisted of 210 subjects. All patients underwent MDCT within 3.1 ± 0.5 days prior to ICA. The study was approved by our institution's scientific and ethical committees and all participating patients gave written informed consent. At admission the pre-test likelihood of CAD was defined using an estimated predicted probability according to American Heart Association (AHA) guidelines for the management of chronic stable angina, based on age, sex, and symptoms [13].

**Table 1:** Baseline Characteristics

	Group 1 Diabetic Patients	Group 2 Control group	p
Number of patients, n	105	105	ns
Gender (male/female),n	92/13	92/13	ns
Age (years), mean ± SD	65.4 ± 9.4	63.3 ± 5.5	ns
BMI (Kg/m2), mean ± SD	28.3 ± 3.4	28. ± 5 4	ns
Serum creatinine (mg/dl), mean ± SD	1.12 ± 0.3	1.04 ± 0.2	ns
CLINICAL HISTORY			
Angina, n (%)	54 (51%)	62 (59%)	ns
Inconclusive stress test, n(%)	56 (53%)	62 (58%)	ns
Valvular disease, n (%)	0 (0%)	2 (2%)	ns
Arrhytmia, n (%)	0 (0%)	2 (2%)	ns
DCM, n (%)	12 (8%)	10 (9.5%)	ns
CARDIOVASCULAR RISK FACTORS			
Diabetes mellitus, n (%)	105 (100%)	0 (0%)	0.01
Insulin dependent, n (%)	12 (11.5%)	-	-
Glycosylated haemoglobin (%)	7.1 ± 2.4%	-	-
Hypertension, n (%)	74 (70%)	63 (60%)	ns
Hypercholesterolemia, n (%)	59 (56%)	45 (43%)	ns
Current smoking, n (%)	31 (30%)	29 (28%)	ns
Family history of CAD, n (%)	21 (20%)	27 (26%)	ns
PRE-TEST LIKELIHOOD OF CAD	51%	52%	ns
β-BLOCKER			
Number of patients, n (%)	66 (63%)	63 (60%)	ns
Dose (mg), mean ± SD	11.2 ± 5.3	11.4 ± 2.5	ns
HEART RATE (bpm), mean ± SD	61.2 ± 9.3	58 ± 4.6	ns
AGATSTON SCORE			
mean ± SD	479 ± 492	356 ± 367	0.01
median (range)	346 (0-1740)	240 (0-1650)	0.01
CAD extension			
0-vessel, n (%)	10 (11%)	18 (13%)	ns
1-vessel, n (%)	28 (27%)	20 (19%)	ns
2-vessel, n (%)	33 (30%)	34 (34%)	ns
3-vessel, n (%)	34 (32%)	33 (34%)	ns

BMI = Body mass Index; CAD = Coronary artery disease; DCM = Dilated cardiomiopathy;

DS = standard deviation.

### Patient preparation for 64-slice MDCT coronary angiography

All patients were connected to an electrocardiographic monitor before the scan and the resting heart rate was monitored continuously. Metoprolol was intravenously administered before MDCT with a titration dose up to 20 mg in all patients with heart rate >65 bpm.

### Scan protocol

Scanning was performed with a 64-slice MDCT scanner (VCT, GE Medical System, Milwaukee, WI) with 64 × 0.625 mm collimation, 330 msec gantry rotation time and 120 Kv tube voltage. Dose modulation was obtained with "electrocardiographic gating" for a maximum gantry delivery between 40% and 80% during the R-R interval and least delivery during the remainder of the cardiac cycle leading to an estimated mean radiation exposure of 14.3 mSv. The "smart prep" scanning was performed in order to obtain a four chamber projection. A bolus of 80 ml of high concentration contrast medium (Iomeron 400 mg/ml, Bracco, Milan, Italy) was administered intravenously at 5 ml/sec, followed by 50 ml of saline injected at the same infusion rate. The scan was initiated according to the bolus-tracking technique.

### MDCT image analysis

The coronary calcium score was assessed with a dedicated software application (CaScore Package - GE Healthcare, Milwaukee, WI) and the overall Agatston score was recorded for each patient. Image data sets were analyzed using volume rendering, multi-planar reconstruction and vessel analysis software packages on a post-processing workstation (Advantage Workstation version 4.2, GE Healthcare, Milwaukee, WI). Images were reconstructed with an effective slice width of 0.625 mm at an increment of 0.4 mm. The causes of impaired image quality were classified as motion artifacts related to the inability to sustain the breath hold, blooming effect due to large calcifications of coronary vessels, slice misalignment related to heart rate variation or premature ventricular beats during the scan, presence of cardiac device and impaired signal/image noise ratio. Coronary artery segments were classified according to the 15-segment AHA classification [14]. All segments with a diameter of at least 1.5 mm at their origin were included. Two independent and blinded readers (D.A., G.P.) classified each vessel segment for the presence of significant stenosis, defined as narrowing of the coronary lumen exceeding 50%. For any disagreement in data analysis between the two readers, consensus agreement was achieved. The CAD burden was defined as the number of coronary segments with significant lesions and of coronary segments with any plaques. Coronary arteries size was evaluated measuring the lumen diameter of origin of each coronary artery segment having more than 1.5 mm in diameter, twice with the fixed window width of 700 Hounsfield units (HU) and window level of 250 HU (WW/WL), which are previously reported [15]. To measure the lumen diameters, the shortest distance between the contrast-agent filled artery contours was evaluated [16]. The time needed for post-processing analysis was recorded.

### Invasive coronary angiography

Conventional ICA was performed with standard technique using 6 Fr catheters. The coronary arteries were classified using the AHA classification [14]. The angiograms were analyzed using a quantitative coronary angiography software (QantCor. QCA, Pie Medical Imaging, Maastricht, the Netherlands) by two interventional cardiologists blinded to MDCT data sets. The severity of coronary stenoses was quantified in 2 orthogonal planes, and a stenosis was classified as significant if the lumen diameter reduction was >50%.

### Statistical analysis

Statistical analysis was performed using the SPSS 13.0 software (SPSS Inc, Chicago, IL). Continuous variables were expressed as mean ± SD, and discrete variables as absolute numbers and percentages. The Student t-test was used to test differences of continuous variables between the two groups, and Chi-square test or Fischer's exact test were used on the basis of the events observed to study differences regarding categorical data. A p value < 0.05 was considered statistically significant. Concerning coronary artery evaluation, the overall feasibility (number of segments evaluable/total number of coronary segments) of the MDCT scan was assessed. An estimation of accuracy (sensitivity, specificity, positive predictive value [PPV] and negative predictive value [NPV]) was calculated on a segment-based model and on a patient-based model, based on a 50% threshold against the standard of ICA findings. On a patient-based analysis, patients with at least 1 detected stenosis >50% in a native coronary artery were classified as "positive". We also perform a segment-based analysis including all segments for analysis with nonevaluable segments censored as "positive" [17]. The 95% confidence interval for these parameters was calculated using the ratio estimator for variance. The diagnostic performance between the two groups was compared using the pairwise McNemar's test. The intra-observer and inter-observer variability for the detection of significant disease on MDCT images were tested with a K test.

## Results

### Baseline characteristics

The two groups were homogeneous in terms of demographic and clinical characteristics with the exception of the presence of DM in all patients of Group 1 (Table 1). In this Group, 12 (11%) patients were insulin-dependent individuals and 95 (89%) were using oral medications. No significant difference was found in b-blockade pre-treatment, heart rate during the scan, CAD extension at ICA and clinical history between the two groups. On the contrary, the calcium score was significantly higher in Group 1 vs. Group 2 (Table 1).

### MDCT feasibility

**Figure 1:**
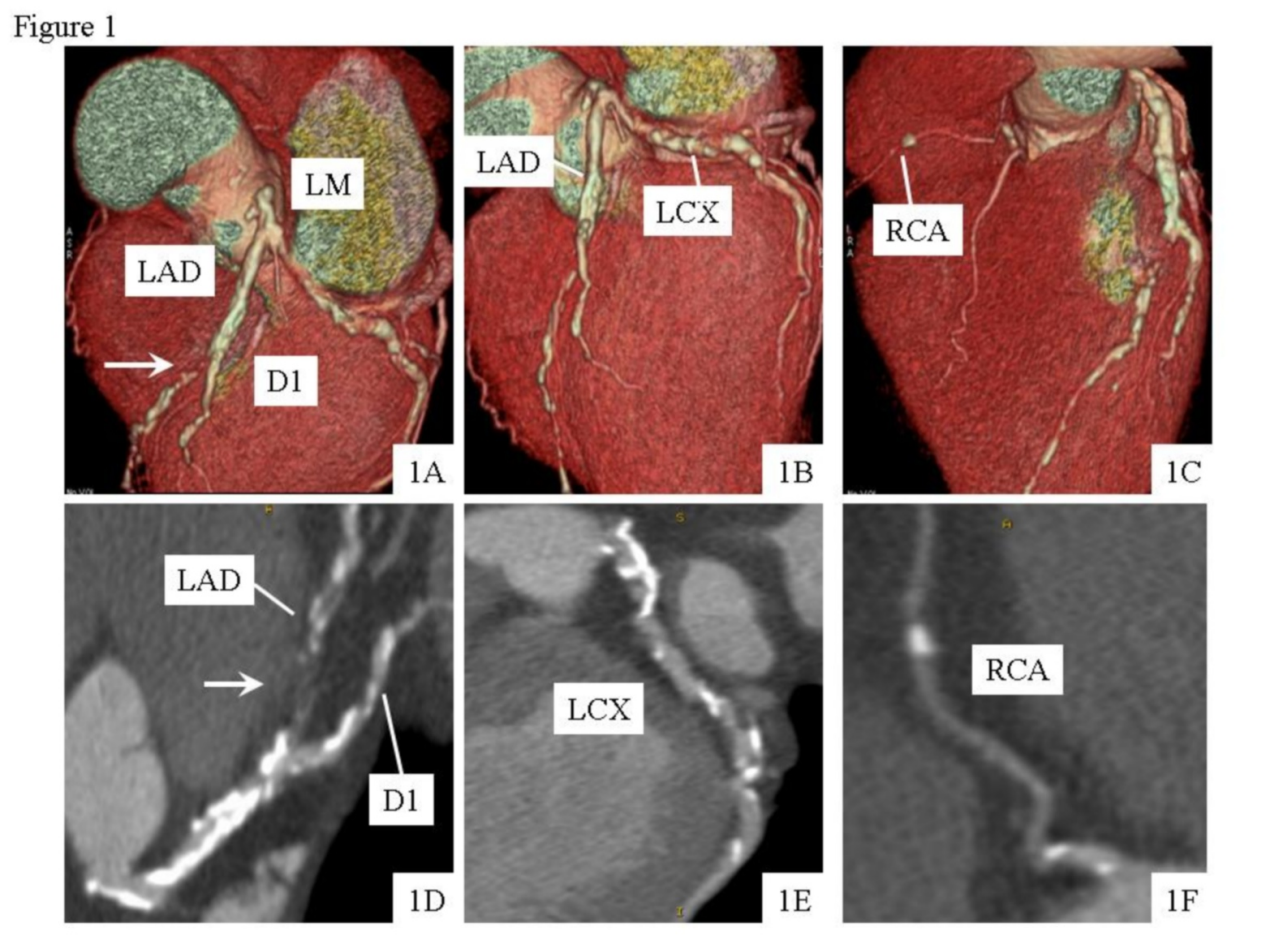
**MDCT volume rendering (upper panels 1A, 1B, 1C) and multiplanar reconstruction (lower panels 1D, 1E, 1F) showing the small coronary size and large coronary calcifications of the epicardial coronary vessels**. The white arrow indicate an occlusion of the middle portion of LAD (1A, 1D). *D1 = first diagonal branch; LAD = left anterior descending coronary artery; LCX = left circumflex artery; LM = left main artery; MDCT = multidetector computed tomography; RCA = right coronary artery.*

The time needed for post-processing analysis was significantly longer in Group 1 (35 ± 18 min) vs. Group 2 (21 ± 14 min, p < 0.01). The overall feasibility was significantly better in Group 2 than in Group 1 (97% vs. 94%, p = 0.0001) due to a significantly lower number of artifacts (39 vs. 81, p < 0.0001). A sub-analysis of the artifacts showed a higher percentage of blooming artifacts due to large calcifications in Group 1 vs. Group 2 (67% vs. 36%, p = 0.0001). In a patient-based model, no significant difference between the two groups was found in the rate of other artifacts (Table 2). Figure 1 shows MDCT result in a diabetic patient and illustrates the two main anatomical features impacting the diagnostic accuracy of this imaging modality: small vessel size and large coronary calcifications.

**Table 2:** Comparison of feasibility and artifact rate in Group 1 and Group 2.

	N°	Feasibility n (%)	Breath n (%)	SM n (%)	Ca n (%)	S/N n (%)	CD n (%)
**GROUP 1 Segments**	1310	1229 (94%)	11 (13%)	13 (16%)	54 (67%)	3 (4%)	-

**GROUP 2 Segments**	1342	1303 (97%)	13 (33%)	10 (26%)	14 (36%)	2 (6%)	-

**P**	-	0.0001	NS	NS	0.0001	NS	-

Breath: artifacts due to breath/chest movement; SM: slice misalignment due to heart rate variability or premature beat during the scan; Ca: blooming artifact due to large calcifications; S/N: impaired image signal/image noise ratio; CD: cardiac device.

### CAD imaging with MDCT

No significant difference was found between the two groups both in terms of number of patients without significant stenoses (16 patients of Group 1 vs. 18 patients of Group 2) and with at least one significant stenosis of the coronary arteries (89 patients of Group 1 vs. 87 patients of Group 2). The extension of CAD was also similar (1-vessel disease: 28 patients in Group 1 vs. 20 patients in Group 2; 2-vessel disease: 30 patients in Group 1 vs. 31 patients in Group 2; 3-vessel disease: 31 patients in Group 1 vs. 36 patients in Group 2). On the contrary, the CAD burden in terms of number of coronary segments with significant lesions (3.1 ± 2.2 vs. 2.5 ± 1.7, p = 0.01) and of coronary segments with coronary plaques (5.3 ± 2.8 in vs. 4 ± 2.4, p = 0.001) was significantly higher in Group 1 than in Group 2.

### Coronary size

As shown in Table 3, the lumen diameter of all coronary segments was significantly lower in Group 1 vs. Group 2, with the exception of 2 segments (proximal left circumflex artery and middle left anterior descending artery).

**Table 3:** Comparison of the coronary artery lumen diameter between Group 1 and Group 2

Segments	Lumen Diameter in Group 1	Lumen Diameter in Group 2	P values
**LM (mean ± SD, mm)**	4,0 ± 0,3	4,2 ± 0,4	**0.0001**

**Proximal LAD (mean ± SD, mm)**	3,5 ± 0,4	3,8 ± 0,2	**0.0001**

**Mid LAD (mean ± SD, mm)**	2,8 ± 0,1	2,8 ± 0,2	**0.30**

**Distal LAD (mean ± SD, mm)**	1,8 ± 0,2	2,3 ± 0,4	**0.0001**

**D1 (mean ± SD, mm)**	1,7 ± 0,3	2,6 ± 0,5	**0.0001**

**D2 (mean ± SD, mm)**	1,6 ± 0,1	2,5 ± 0,5	**0.0001**

**Proximal LCX (mean ± SD, mm)**	3,0 ± 0,1	3,0 ± 0,4	**0.56**

**Mid LCX (mean ± SD, mm)**	2,5 ± 0,3	2,8 ± 0,5	**0.008**

**Distal LCX (mean ± SD, mm)**	2,4 ± 0,2	2,3 ± 0,5	**0.001**

**M1 (mean ± SD, mm)**	2,5 ± 0,4	2,7 ± 0,5	**0.0001**

**M2 (mean ± SD, mm)**	1,8 ± 0,5	2,4 ± 0,4	**0.0001**

**Proximal RCA (mean ± SD, mm)**	3,0 ± 0,2	3,9 ± 0,5	**0.0001**

**Distal RCA (mean ± SD, mm)**	2,8 ± 0,2	3,4 ± 0,5	**0.0001**

**PLA (mean ± SD, mm)**	2,5 ± 0,2	2,8 ± 0,4	**0.0001**

**PDA (mean ± SD, mm)**	2,6 ± 0,2	3,0 ± 0,4	**0.0001**

D1 = First diagonal branch; D2 = Second diagonal branch; LM = left main;

LAD = left anterior descending; LCX = left circumflex; M1 = First marginal branch;

M2 = Second marginal branch; PLA = Postero-lateral artery;

PDA = Posterior descending artery.

### MDCT accuracy in a segment-based model Group 1

The consensus between MDCT and ICA in classifying the coronary stenoses as significant was achieved in 309 out of 1229 segments and as angiographically normal in 920 out of 1229 segments. Overall, 69 lesions were underestimated by MDCT and 218 segments were incorrectly graded as significantly stenotic. Sensitivity was 76%, specificity 90% and overall accuracy 87% (Table 4). The Kappa value for the detection of significant coronary artery disease was 0.78 for intra-observer agreement and 0.74 for inter-observer agreement.

**Table 4:** Diagnostic accuracy of MDCT for the detection of significant (>50%) coronary stenosis using a segment-based model in Group 1 (Segments for analysis only).

Segments	N	TN	TP	FN	FP	Se	Sp	NPV	PPV	Accuracy
**LM**	99	85	1	7	6	13%	93%	92%	14%	87%
**Proximal LAD**	99	44	43	0	12	100%	79%	100%	78%	88%
**Mid LAD**	93	40	38	7	8	84%	83%	85%	83%	84%
**Distal LAD**	98	86	6	4	2	60%	98%	96%	75%	94%
**D1**	80	48	15	6	11	71%	81%	89%	58%	79%
**D2**	58	52	1	1	4	50%	93%	98%	20%	91%
**Proximal LCX**	101	65	22	3	11	88%	86%	96%	67%	86%
**Mid LCX**	95	70	16	4	5	80%	93%	95%	76%	91%
**Distal LCX**	92	84	3	2	3	60%	97%	98%	50%	95%
**M1**	76	54	8	7	7	53%	89%	89%	53%	82%
**M2**	17	14	2	1	0	67%	100%	93%	100%	94%
**Proximal RCA**	91	47	25	11	8	69%	85%	81%	76%	79%
**Distal RCA**	89	44	28	8	9	83%	78%	85%	76%	83%
**PLA**	64	52	6	2	4	75%	93%	96%	60%	91%
**PDA**	77	66	2	7	2	22%	97%	90%	50%	88%
**All segments**	1229	851	218	69	91	76% (72-82)	90% (88-92)	93% (91-94)	71% (65-73)	87% (86-90)

D1 = First diagonal branch; D2 = Second diagonal branch; FN = false negative; FP = false positive;

LM = left main; LAD = left anterior descending; LCX = left circumflex; M1 = First marginal branch;

M2 = Second marginal branch; NPV = negative predictive value; PLA = Postero-lateral artery;

PDA = Posterior descending artery; PPV = positive predictive value; Se = Sensitivity;

Sp = Specificity; TN = true negative; TP = true positive;

### Group 2

The presence of significant stenoses was correctly detected in 251 segments and correctly excluded in 994 segments. Twenty-one significant lesions were missed by MDCT and 37 stenoses rated as significant by MDCT were not confirmed by ICA. Sensitivity was 92%, specificity 96% and accuracy 96% (Table 5). The Kappa value for detection of significant coronary artery disease was 0.82 for intra-observer agreement and 0.78 for inter-observer agreement.

**Table 5:** Diagnostic accuracy of MDCT for the detection of significant (>50%) coronary stenosis using a segment-based model in Group 2 (Segments for analysis only).

Segments	N	TN	TP	FN	FP	Se	Sp	NPV	PPV	Accuracy
**LM**	104	99	4	1	0	80%	100%	99%	100%	99%
**Proximal LAD**	94	46	45	1	2	98%	96%	98%	96%	97%
**Mid LAD**	91	44	42	1	4	98%	92%	98%	91%	94%
**Distal LAD**	99	86	10	2	1	83%	99%	98%	91%	97%
**D1**	86	66	19	0	1	100%	98%	100%	95%	99%
**D2**	30	27	3	0	0	100%	100%	100%	100%	100%
**Proximal LCX**	96	70	17	3	6	85%	92%	96%	74%	91%
**Mid LCX**	94	72	18	2	2	90%	97%	97%	90%	96%
**Distal LCX**	101	89	8	2	2	80%	98%	98%	80%	96%
**M1**	89	72	9	2	6	82%	92%	97%	60%	91%
**M2**	24	19	2	0	3	100%	86%	100%	40%	88%
**Proximal RCA**	99	66	28	0	5	100%	93%	100%	85%	95%
**Distal RCA**	96	66	25	3	2	89%	97%	96%	93%	95%
**PLA**	100	85	12	1	2	92%	98%	99%	86%	97%
**PDA**	100	87	9	3	1	75%	99%	97%	90%	96%
**All segments**	1303	994	251	21	37	92% (87-94)	96% (94-97)	98% (96-99)	87% (86-89)	96% (93-97)

D1 = First diagonal branch; D2 = Second diagonal branch; FN = false negative; FP = false positive;

LM = left main; LAD = Left anterior descending; LCX = left circumflex; M1 = First marginal branch;

M2 = Second marginal branch; NPV = Negative predictive value; PLA = Postero-lateral artery;

PDA = Posterior descending artery; PPV = Positive predictive value; Se = Sensitivity;

Sp = Specificity; TN = True negative; TP = True positive;

### Comparison of MDCT diagnostic accuracy in a segment-based model

All the diagnostic parameters (sensitivity, specificity, NPV, PPV and accuracy) were significantly (p = 0.001) higher in Group 2 than in Group 1, either including only the evaluable segments or all segments for analysis with those nonevaluable censored as positive (Table 6).

**Table 6:** Comparison of the diagnostic accuracy of MDCT for the detection of significant (>50%) coronary stenosis between Group 1 and Group 2.

SEGMENT-BASED MODEL - SEGMENTS FOR ANALYSIS ONLY										
**Group**	**N°**	**TN**	**TP**	**FN**	**FP**	**Se**	**Sp**	**NPV**	**PPV**	**Accuracy**

**Group 1**	1229	851	218	69	91	76% (72-82)	90% (88-92)	93% (91-94)	71% (65-73)	87% (86-89)

**Group 2**	1303	994	251	21	37	92% (87-94)	96% (94-97)	98% (96-99)	87% (86-89)	96% (93-97)

**P**	-	-	-	-	-	0.001	0.001	0.001	0.001	0.001

**SEGMENT-BASED MODEL - ALL SEGMENTS FOR ANALYSIS WITH NON EVALUABLE SEGMENTS "POSITIVE"**										

**Group**	**N**	**TN**	**TP**	**FN**	**FP**	**Se**	**Sp**	**NPV**	**PPV**	**Accuracy**

**Group 1**	1310	851	243	69	147	78% (73-82)	85% (83-87)	92% (91-94)	62% (57-65)	83% (81-85)

**Group 2**	1342	994	251	21	76	92% (89-95)	93% (91-94)	98% (97-99)	77% (81-92)	93% (91-94)

**P**	-	-	-	-	-	0.001	0.001	0.001	0.001	0.001

**PATIENT-BASED MODEL - SEGMENTS FOR ANALYSIS ONLY**										

**Group**	**N°**	**TN**	**TP**	**FN**	**FP**	**Se**	**Sp**	**NPV**	**PPV**	**Accuracy**

**Group 1**	105	3	87	6	9	94% (89-99)	33% (0-51)	25% (0-90)	91% (85-100)	86% (82-88)

**Group 2**	105	15	83	4	3	95% (91-100)	83% (66-100)	79% (61-97)	97% (93-100)	93% (89-98)

**P**	-	-	-	-	-	NS	0.01	0.001	NS	0.05

FN = False negative; FP = False positive; NPV = negative predictive value; PPV = positive

predictive value; Se = Sensitivity; Sp = Specificity; TN = true negative; TP = true positive.

### Comparison of MDCT diagnostic accuracy in a patient-based model

In Group 1, 87 out of 93 patients with significant stenoses in at least one coronary segment at ICA were correctly identified by MDCT (sensitivity 94%). Six patients were missed by MDCT. Moreover, significant CAD was correctly ruled out by MDCT in 3 patients, but in 9 cases a stenosis was diagnosed as significant by MDCT and was found to be less than 50% at ICA (specificity of 33%). Specificity, NPV and accuracy were significantly higher in Group 2 (83%, 79% and 93%, respectively) than in Group 1 (33%, 25% and 86%, respectively) (Table 6).

## Discussion

The main findings of this study indicate that the non-invasive detection of significant coronary artery stenoses with 64-slice MDCT in DM patients as compared to their non-diabetic counterparts with similar clinical characteristics and suspicion of CAD requires a longer time for post-processing analysis and has lower feasibility and diagnostic accuracy. Indeed, the overall feasibility of MDCT evaluation of the coronary tree was significantly lower in the DM Group than in non-diabetic patients, due to a significantly higher number of artifacts. In agreement with previous studies that demonstrated a higher rate of extensive coronary artery calcifications in diabetics [3,6,11], our feasibility sub-analysis showed that blooming artifacts due to large calcifications were significantly more frequent in Group 1 as compared to Group 2. A very recent study of Chu et al. confirmed that calcified plaques are the most common type of coronary plaque in diabetics [18]. Of note, no difference was found in the present study between the two Groups in the rate of other artifacts that can affect MDCT accuracy. This was likely due to similar demographic and clinical characteristics and heart rate during the scan in the two Groups.

Our study shows that the diagnostic performance of MDCT in DM patients with suspected CAD is significantly lower compared to non-diabetic patients with similar clinical characteristics and also lower than that reported by previous studies that included patients without DM [4]. Indeed, in our DM population, 69 lesions were underestimated by MDCT and 218 segments were incorrectly graded as significantly stenotic. In a segment-based model, the overall sensitivity was 76%, with a value of only 13% for the left main coronary artery, while specificity and accuracy were 90% and 87%, respectively. Thus, all parameters were significantly lower in the DM Group than those found in the non-diabetic Group because the former had a lower prevalence of false negative (21 segments) and showed mainly false positive (37 segments). Similarly, in a patient-based model the diagnostic performance of MDCT in the DM Group was less than satisfactory, mainly because 9 patients were diagnosed with MDCT as affected by significant CAD that was not confirmed by ICA. This explains the significantly lower specificity, NPV and accuracy found in DM patients as compared to their non-diabetic counterparts. The worse diagnostic accuracy found in DM patients is likely due to several factors. First, DM is associated with more prevalent and extensive coronary calcifications that impede the correct visualization of the coronary lumen. Indeed, accumulation of calcium in the arterial wall of patients affected by DM is not limited to the subintimal space, but often extends in the medial layer [5]. A very recent study of Maffei et al. confirmed that coronary plaque burden and coronary calcium score are higher in diabetic vs. non-diabetic patients [19]. The calcium burden affects not only MDCT feasibility but also the quantification of the coronary stenosis, sometimes leading to an overestimation of the lesion severity [20]. Second, compared to non-diabetic individuals, DM patients have a more extensive plaque burden, as shown in several previous studies [6,21] and confirmed by our results, which has a strong influence on MDCT diagnostic accuracy [22]. Third, the small coronary size and lumen area, typical of DM patients [10,23] and confirmed in our study, cause difficulties detecting focal lesions and differentiating between significant and non-significant stenoses, since the small coronary lumen dimension is proximal to the imaging technique resolution.

Finally, it is noteworthy that the large CAD burden and the difficulty to correctly evaluate a coronary artery with massive wall calcifications and small lumen diameter increased significantly the time needed for post-processing analysis of the MDCT images in diabetic compared to non-diabetic patients. These shortcomings underscore the need to develop further refinement of MDCT technology to overcome the problems related to the CAD features of DM.

### Clinical Implications

Our study demonstrates that in a high-risk population with unknown CAD, as in the case of our DM patients, MDCT often find severe coronary atherosclerosis, both in terms of the number of patients with significant multi-vessel disease and atherosclerotic burden. This finding is in agreement with those of previous studies with 64-slice MDCT that indicated a higher prevalence of any type of coronary plaques and of significant coronary stenoses and a larger plaque burden in asymptomatic diabetics compared to non-diabetic patients [6,21]. These results support the concept that MDCT coronary angiography is an excellent noninvasive technique for early identification of significant CAD in high-risk patients with inconclusive or unfeasible noninvasive stress test results [24]. Thus, it may represent a valuable tool for early detection of CAD to risk-stratify DM patients with the aim of differentiating those who are in need of coronary revascularization from those who may benefit from aggressive medical management. In this regard, despite the lower overall diagnostic accuracy in the DM population, 87 out of 93 patients with a significant stenosis in at least one coronary segment at ICA were correctly identified by MDCT. According to the patient-based model, this translated in a sensitivity of 94%. On the hand, the number of DM patients in whom CAD severity was overestimated is one of the clear limitations of MDCT that was underlined by this study. Indeed, significant CAD was correctly ruled out by MDCT in 3 patients, but in 9 cases a stenosis was diagnosed as significant by MDCT and was found to be less than 50% at ICA, translating in a specificity of only 33%. This low specificity in diabetics should be taken in account when interpreting MDCT results because may lead the physician to subject several patients to an ICA that is not clinically needed for them because CAD is diffuse but subcritical. Recently, a high-definition MDCT scanner with improved in-plane spatial resolution (230 μm) and the ability to reconstruct images using a novel applied statistical iterative reconstruction algorithm has been introduced in the clinical practice [25]. It's conceivable that the higher spatial resolution would be particularly useful for the assessment of coronary vessels with large calcifications as in the case of DM patients.

### Study limitations

We describe the results of a single-center study and the number of enrolled patients was relatively small. To date, however, this study represents the largest cohort of DM patients in whom diagnostic accuracy of MDCT was compared to non-diabetics subjects. Moreover, the two groups were well matched in terms of demographic characteristics and coronary risk factors other than DM.

## Conclusions

Although MDCT has high sensitivity for early identification of significant CAD in diabetics, its diagnostic performance is significantly reduced in these patients as compared to non-diabetics with similar clinical characteristics.

## Abbreviations List

CAD: coronary artery disease; DM: diabetes mellitus; ICA: invasive coronary angiography; MDCT: multidetector computed tomography; NPV: negative predictive value; PPV: positive predictive value

## Competing interests

The authors declare that they have no competing interests.

## Authors' contributions

DA, MP, PA, GB, AB and CF contributed in the conception and design of the study and in drafting of the manuscript. SM, SC, LA, and MC contributed in the collection and in the analysis and interpretation of MDCT data. GP, DA and AA performed and reviewed all MDCT examinations. All authors read and approved the final manuscript.
